# Design and Implementation of an Off-Grid Smart Street Lighting System Using LoRaWAN and Hybrid Renewable Energy for Energy-Efficient Urban Infrastructure

**DOI:** 10.3390/s25175579

**Published:** 2025-09-06

**Authors:** Seyfettin Vadi

**Affiliations:** Department of Electrical and Electronics Engineering, Faculty of Technology, Gazi University, Ankara 06500, Turkey; seyfettinvadi@gazi.edu.tr

**Keywords:** off-grid energy systems, SCADA interface, LoRaWAN, remote monitoring and control, P&O algorithm, MQTT

## Abstract

**Highlights:**

**What are the main findings?**

- Ensuring energy efficiency through the use of the P&O MPPT algorithm
- The integration of a PV-battery hybrid system for a smart street lighting system
- The use of internet-free LoRaWAN communication for a remote monitoring and control system

**What is the implication of the main finding?**

- The primary objective of this study is to present a design for a street lighting system based on LEDs, which is hybrid-powered by solar energy and batteries, thereby making it independent of the grid.
- It focuses on reducing energy consumption during times of low demand, managing energy according to the potential of energy sources, and enhancing system reliability by enabling monitoring and control of the entire system through the LoRaWAN communication method.

**Abstract:**

The growing demand for electricity and the urgent need to reduce environmental impact have made sustainable energy utilization a global priority. Street lighting, as a significant consumer of urban electricity, requires innovative solutions to enhance efficiency and reliability. This study presents an off-grid smart street lighting system that combines solar photovoltaic generation with battery storage and Internet of Things (IoT)-based control to ensure continuous and efficient operation. The system integrates Long Range Wide Area Network (LoRaWAN) communication technology for remote monitoring and control without internet connectivity and employs the Perturb and Observe (P&O) maximum power point tracking (MPPT) algorithm to maximize energy extraction from solar sources. Data transmission from the LoRaWAN gateway to the cloud is facilitated through the Message Queuing Telemetry Transport (MQTT) protocol, enabling real-time access and management via a graphical user interface. Experimental results demonstrate that the proposed system achieves a maximum MPPT efficiency of 97.96%, supports reliable communication over distances of up to 10 km, and successfully operates four LED streetlights, each spaced 400 m apart, across an open area of approximately 1.2 km—delivering a practical, energy-efficient, and internet-independent solution for smart urban infrastructure.

## 1. Introduction

One of the United Nations’ Sustainable Development Goals, accessible and clean energy, is related to energy access that closely affects daily life. It encompasses ensuring that everyone has access to energy at an affordable price. The aim is to make sustainable and clean energy universally accessible. Additionally, it is aimed to create a reliable energy system that supports economic development. The initiative aims to strengthen global cooperation and attract investment in energy infrastructure and clean energy solutions. It focuses on research and development in renewable energy, energy efficiency, and cleaner fossil fuel technologies. The target is to achieve these goals by 2030. Sustainable development seeks to protect both present and future needs by balancing environmental, economic, and social priorities. Efforts should promote energy efficiency, use renewable energy sources, and align with carbon reduction and green environment principles [1].

Addressing global energy challenges requires international cooperation and large-scale strategies. At the same time, local and technology-driven solutions, such as smart street lighting systems, are essential for improving energy efficiency and promoting sustainable urban development. Traditional street lighting systems account for a substantial portion of urban energy consumption, leading to high operational costs and significant environmental impacts. In response to these challenges, smart street lighting systems employing Light-Emitting Diode (LED) technology have emerged as a promising solution for enhancing energy efficiency, minimizing maintenance expenses, and promoting environmental sustainability [2]. As urbanization accelerates and the smart city paradigm gains global momentum, the demand for innovative technologies that support energy conservation and sustainable development continues to rise. Street lighting systems play a crucial role in smart city infrastructures by enhancing both the quality of life and economic sustainability through the integration of advanced technologies. Within this framework, the Internet of Things (IoT) facilitates robust interconnectivity among devices, sensors, and networks, forming the backbone of intelligent urban systems. Moreover, IoT-enabled control mechanisms enable real-time and adaptive regulation of lighting intensity, thereby optimizing energy usage while enhancing public safety.

When examining the studies conducted in the literature, various problems are observed in street lighting systems, such as high energy consumption of the lamps, low system efficiency, the inability to know the type or location of lamp failures in advance, fixed lamp brightness, high cost, excessive need for physical inspection and maintenance teams, high energy consumption, greenhouse gas emissions, and carbon footprint, low safety level, delayed detection of faults and errors, long repair times, and finally, high light pollution [3,4,5,6]. A holistic approach to solving these problems has not been presented.

Today, technology is developing at a rapid pace. This change is particularly evident in the field of electrical and electronic engineering. New technologies are emerging as new products, while also being applied to enhance existing ones. The emerging LED technology can be integrated with IoT technology to create smart systems. Thus, existing or traditional systems are brought to an advantageous position. Innovative independent street lighting systems provide significant energy savings. Therefore, the imminent depletion of fossil fuel resources and their continuously rising prices due to inflation have made advanced energy management and monitoring strategies, aimed at reducing energy consumption and transitioning to a low-carbon economy by 2050, inevitable. Approximately 80% of the electricity produced is used to meet the needs of cities, with 60% of this energy consumed by street lighting systems that operate continuously throughout the night. This underscores the importance of energy conservation as a critical priority for smart cities, particularly through the adoption of LED-based intelligent lighting systems as efficient alternatives to conventional incandescent and fluorescent lamps, leading to substantial energy savings [7,8].

Additionally, street lighting systems are crucial for enhancing the quality of life in urban areas. In addition to increasing safety on roads or in public places, it contributes to economic development by extending the time people spend outdoors at night. However, the excessive number of these systems leads to high energy consumption and increased carbon dioxide emissions, exacerbating the greenhouse effect. In conclusion, enhancing the efficiency of lighting systems by integrating them with new technologies is a crucial issue for the national economy and cities. Several methods in the literature aim to enhance the efficiency of street lighting systems [9].

The first of these methods involves replacing the traditional high-pressure sodium (HPS) lamp with an LED lamp. The LED lamp consumes less energy compared to the HPS lamp. The long lifespan of LED lamps and the decreasing cost due to technological advancements contribute to their preference. Considering the large number of lamps in street lighting, the scale of energy efficiency becomes apparent. Additionally, HPS lamps heat up more than LED lamps and use 40% of the provided energy for lighting [10,11,12,13].

As a second method, LED lamps offer even greater efficiency when combined with light-dependent resistors (LDR), infrared sensors (IR), or ultrasonic sensors in LED-based street lighting systems. A wireless sensor network, comprising sensors, microcontrollers, and wireless communication modules, controls the lamps’ on-off operation [14,15,16,17,18].

As a third method, IoT technology enables remote monitoring and management of the lighting system. LoRAWAN technology is also a new wireless communication method that provides updates by being used with products available in the market due to its advantages. This technology, particularly with lighting systems, significantly contributes to smart applications, including smart grids and smart cities. For IoT-based street lighting systems, cloud-based platforms have been proposed using wireless communication technologies such as ZigBee [14], WiMAX [19], GSM [20,21,22], LoRa [23,24], and NB-IoT [25].

Recent research has introduced several techniques for hardware-based energy optimization at IoT systems [26,27]. Various optimization algorithms have been proposed to reduce overall energy consumption with promising results. For instance, Raval et al. employed the block cipher PRESENT on lightweight, compact hardware, achieving an estimated 16% reduction in energy usage. Similarly, Khan et al. proposed a decentralized scheduling mechanism for status updates in IoT systems, which, through optimized software channel utilization, reduced energy consumption by approximately 15%. Zhai and Zhang developed a specialized power allocation strategy for wireless networks with massive IoT devices, reporting a 17% reduction in energy usage by efficiently scheduling sensor activation and deactivation [28]. Energy consumption is reduced, and system efficiency is increased thanks to the hardware structure and software algorithms developed in IoT applications. Specifically, new-generation updates are made to existing systems, such as street lighting systems, to make the systems behave more intelligently.

In smart street lighting systems, energy efficiency and sustainability are other vital aspects. For this, it is primarily essential that the type of energy source is renewable. Solar cells are utilized as an alternative energy source in smart, independent street lighting systems that incorporate LED light lamps [29,30,31]. In their study, Mohanty and colleagues address the design and development of a smart street lighting management system. The system’s energy needs are met through hybrid energy harvesting (solar energy and solar thermal energy), and energy storage is achieved using environmentally friendly lithium-ion capacitors instead of batteries. The energy obtained from solar power has been stored in batteries during the day, allowing the streetlights to turn on when lighting is needed [32]. Thus, using renewable energy sources has enabled the development of environmentally friendly and next-generation applications [33,34]. SARR and colleagues have implemented a LoRaWAN-based street lighting system that automatically turns on or off based on sunlight and periodically collects data about electrical poles to plan maintenance tasks. In the proposed application study, remote lighting control is provided with a network server to adjust light and light intensity duration to minimize energy costs without compromising lighting quality. The application dynamically adjusts the light based on time, weather, motion, and lighting levels. The user interface also monitors the battery levels, accumulated dust level, and solar panel status. Thus, the system’s efficiency is continuously monitored [35].

LED street lighting technology is increasingly used daily due to its characteristics, such as the future depletion of fossil fuels used in energy production, reduced carbon emissions, improved energy efficiency, long lifespan, and developability [36]. This increase causes disruptions in situations such as intervention in case of a malfunction, detection of the malfunction point, determination of the type of malfunction, and estimation of the lifespan. The increasing need for labor also incurs additional costs [37]. Eliminating these negative situations and the importance of using developing technology alongside existing systems are highlighted. Thanks to advancing technology, a new generation of products emerges when streetlights are combined with the Internet of Things. Thus, thanks to IoT, devices can connect with other systems and devices over the internet or wireless communication methods, allowing them to collect data, be monitored, and exchange information. This new generation of communication approach facilitates interactions between humans and devices, or between them, through wireless communication technologies such as Wi-Fi, GSM, and Zigbee [38,39].

It is necessary to connect the lamp posts to a monitoring and control system via a network infrastructure to facilitate the management of streetlights. Considering the static configuration of smart street lighting systems, wireless networks are a viable option. Different routing policies are used within the network to facilitate message exchange (commands or information requests) between smart street poles. As a result, it has the ability to transmit gateway commands or information requests individually to smart streetlights, a selected group of poles, or all smart poles connected to the communication network. Additionally, data exchange between the gateway (network coordinator), the monitoring system, and users’ smartphones is facilitated through a WiFi/3G/4G/LiFi communication network. Most technological solutions rely on the use of WiFi by smartphones. This situation is increasing the constant demand for higher data rates. Network operators are having to deploy new technologies like 5G to solve this problem. Additionally, mobile phone base stations may have some negative effects on health. In addition to wireless technologies like WiFi, ZigBee, RF, and 2G/3G/4G, wired options such as Power Line Communication (PLC) or Ethernet cables can also be used to connect streetlights. The system architecture uses both remote and local communication networks. While the local network facilitates information sharing between smart poles, the remote network enables communication between smart poles and the remote monitoring unit. This setup enables data collection, including the power consumption of the monitoring unit, traffic conditions across the street, and weather data [40]. Communication between smart streetlights is based on the ZigBee wireless process, which is accepted as a universal and worldwide standard for wireless device connectivity. Zigbee technologies offer seamless integration with IoT software using Zigbee gateways. However, these technologies are used in small-scale studies due to the small data size and communication distance [41]. However, the use of RF communication is decreasing due to the high bandwidth demand. This situation is leading to a shift toward wireless technology areas in alternative spectrum bands. Additionally, wireless RF networks exacerbate the problem of increasing exposure to electromagnetic fields in today. This situation can cause concern among people [42]. Power Line Communication (PLC) is used for High-Speed Data Distribution in a network environment. When combined with VLC, PLC can leverage the main infrastructure for transport and allow for wireless connectivity between devices and the internet. VLC technology—LiFi (Light Fidelity) is known as an internet access point for LEDs. LiFi represents the future of mobile internet thanks to its low cost and higher efficiency. It is seen as a competitor to WiFi as a fundamental technology for IoT. However, measurements are taken only when the transmitter and receiver are aligned and the distance between them is less than 1 m. Therefore, amplifiers are needed when LiFi is integrated into a real-world scenario. Additionally, since the LiFi communication system is used with mobile internet, it constantly generates variable costs each month [43]. LiFi technology is used in on-grid systems because it provides communication over the power grid. This technology is not used in island mode energy systems.

Internet of Things (IoT) based systems, wireless technologies such as Wi-Fi, Bluetooth, and Zigbee are utilized, and these technologies have limitations in terms of the number of connected devices, range, and energy consumption [43]. Low-Power Wide Area Network (LPWAN) technologies have gained popularity due to their long ranges, energy efficiency, and low costs. Table 1 presents the characteristics of various wireless technologies, including BLE [44,45], ZigBee [46], Wi-Fi [47], SigFox [48], LoRa [49], and NB-IoT [50], for IoT applications. LoRa is the most widely adopted Low Power Wide Area Network (LPWAN) technology globally, surpassing alternatives such as Sigfox and NB-IoT in deployment across numerous countries. This study selected LoRa due to its extended communication range, which is particularly advantageous for long-distance data transmission. Furthermore, LoRa offers superior receiver sensitivity and lower energy consumption than other LPWAN technologies, making it an efficient and reliable choice for energy-constrained IoT applications [50,51]. Additionally, LoRa’s network protocol, LoRaWAN, is an Open Standard, unlike Sigfox and NB-IoT, which facilitate the widespread use of this technology. In addition, LoRaWAN has lower equipment and installation costs [52].

A comparison table of wireless technology methods, in terms of data speed, range, and power efficiency, is provided in Table 1. Accordingly, considering the data size, energy consumption decreases as the distance increases. LoRaWAN, with its low energy consumption, wide range, and cost-effective advantages, is ideal for applications such as agriculture that require broad coverage and low data rates. However, alternatives such as Wi-Fi, Zigbee, or Bluetooth may be a better choice for high data rates, low latency, and short-range applications. The most significant advantage of LoRaWAN over Sigfox is its customizable network architecture and extensive ecosystem support. LoRaWAN has a low data transfer rate, with a maximum of a few kilobits per second (kbps). This is not suitable for applications that require high bandwidth. In contrast, technologies like WiFi or LTE offer much higher data speeds, but their energy consumption and coverage area are limited. Methods like ZigBee also offer low energy consumption, but cannot reach the extended range of LoRaWAN [52]. LPWAN is particularly suitable for IoT applications that require transmitting only small amounts of data. GPRS, NB-IoT, SIGFOX, LoRa, LoRaWAN, WiFi, etc. Many LPWAN technologies have emerged with characteristics such as licensed and unlicensed frequency bandwidth, various modulation types, differing sensitivities, and diverse transmission ranges [53].

**Figure 1:**
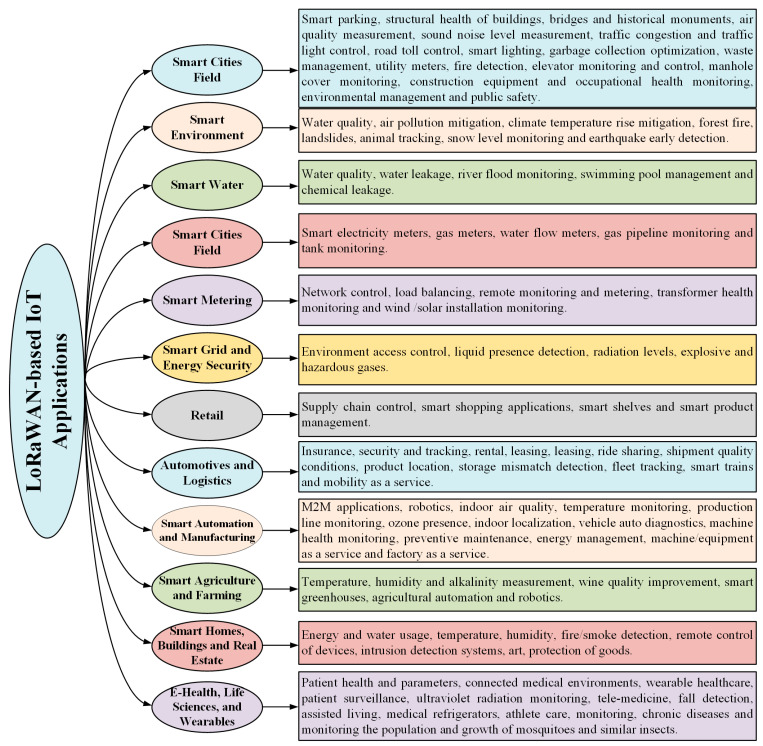
Application studies in the literature using the LoRaWAN communication method.

IoT applications impose stringent requirements, including extended transmission range, low data throughput, minimal energy consumption, and economic feasibility. Conventional short-range wireless technologies such as ZigBee and Bluetooth fall short in meeting these demands, particularly in scenarios requiring wide-area coverage. Although cellular-based communication technologies like 2G, 3G, and 4G offer broader coverage, their high energy demands render them inefficient for most IoT deployments. Consequently, these limitations have driven the development of LoRaWAN, a low-power wide-area network (LPWAN) technology specifically designed to address the unique constraints of IoT systems. LoRaWAN is gaining popularity in industrial and research communities due to its low-power, long-range, and low-cost communication features. It provides communication ranges of 10–40 km in rural areas and 2–5 km in urban areas. However, it is highly energy-efficient (i.e., with a battery life of 10+ years) and cost-effective. Critical use cases for LoRaWAN networks include smart cities, smart street lighting, asset tracking and condition monitoring, supply chain management, electrically powered smart grids, water and gas measurement, smart agriculture, and livestock monitoring in field conditions [53]. Figure 1 presents the commonly used applications of the LoRaWAN wireless communication method as reported in the literature.

**Figure 2:**
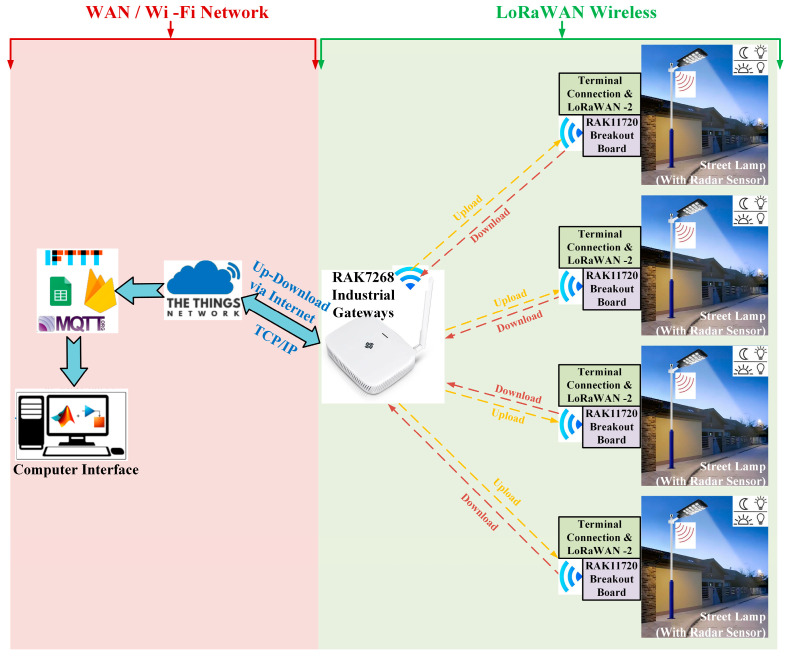
Block diagram of a LoRaWAN-based street lamp.

The LoRaWAN communication method was chosen for this study because it offers long range, low energy consumption, low installation cost, and high data capacity compared to other methods. The primary objective of this study is to present a design for a street lighting system based on LEDs, which is hybrid-powered by solar energy and batteries, thereby making it independent of the grid. Thanks to the developed energy management algorithm, the battery is charged to its maximum capacity with solar energy during the day, and this stored energy is used in street lighting in a controlled manner at night. The MPPT algorithm was used to achieve maximum solar energy production. It focuses on reducing energy consumption during times of low demand, managing energy according to the potential of energy sources, and enhancing system reliability by enabling monitoring and control of the entire system through the LoRaWAN communication method. The block schema of the design and application of a remote monitoring system based on LoRaWAN for modern street lighting systems in innovative applications is shown in Figure 2. In this study, a LoRaWAN wireless communication module has been installed on each streetlight. These modules wirelessly transmit the energy and circuit parameters of the streetlight to the gateway. The transmitted data is stored in the cloud via the gateway and transferred to the user interface through MQTT. The control parameter sent from the user interface also controls the streetlight similarly.

The energy supply for each street lamp has been created independently of the grid. For this purpose, the management of a DC bar, which utilizes solar energy and a battery, is provided to meet the energy needs of the street lamp. The energy produced by the solar panel during the day is maximized using the P&Q-MPPT algorithm, ensuring the battery is charged. This research proposes an efficient off-grid street lighting solution based on P&O-MPPT using LoRaWAN communication without internet access. This solution is scaled to a four-street lamp setup. Thanks to the energy method system created, the energy demand of the street lamp is provided according to the condition of the energy sources.

The primary contributions of this study are fourfold. First, the proposed system enhances energy efficiency and reduces overall energy consumption through optimized utilization of available resources. Second, it introduces an integrated energy harvesting subsystem that combines solar energy with battery storage, thereby ensuring reliable and continuous operation. Third, the study provides a cost-effective and environmentally sustainable solution that is adaptable to the requirements of both urban and rural contexts. Finally, by incorporating IoT-based monitoring and control mechanisms, the system achieves significant energy savings while simultaneously improving the quality and reliability of lighting.

The rest of this paper is structured as follows. Section 2 introduces the fundamental characteristics of the LVDC Energy Bus. Section 3 details the development of the MPPT algorithm and the control strategy implemented using a microcontroller, along with the associated hardware and software components. Section 4 discusses server-side considerations, particularly with respect to cloud-based implementations. Finally, Section 5 presents the experimental results obtained from the LoRaWAN-based smart street lighting system.

## 2. Methodology

### 2.1. LVDC Energy Bus Configuration

**Figure 3:**
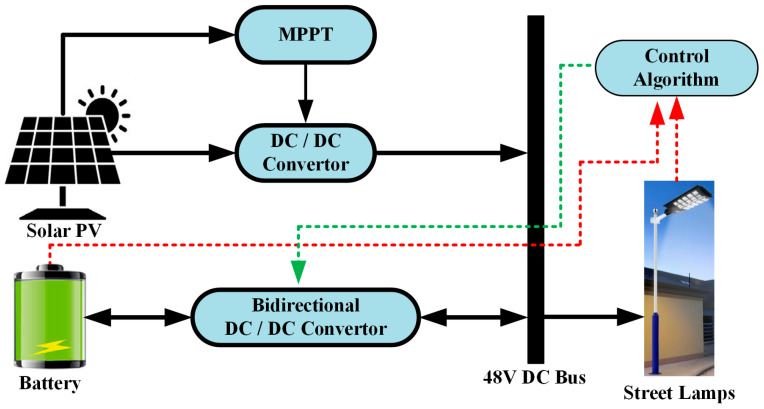
LVDC Energy bus with solar energy and battery for street lamp.

This section describes the design of a low-voltage DC (LVDC) power distribution system for PV and battery-based streetlights. The operating modes (Mode 1–5) are presented in sufficient detail and in a logical manner, explaining how the system responds to changing energy production and demand conditions. Figure 3 illustrates the low-voltage energy system for the proposed streetlight, comprising solar energy and a battery. The bus voltage level is 48 V DC. The energy structure of the system consists of solar energy, a battery storage system, and a controller as its primary components. A DC-DC boost converter equipped with a P&O-MPPT controller creates an interface between the solar energy and the DC bus. This control technique has been preferred in this study due to its simple design and the small number of components. A 12-volt, 9-amp-hour battery was used for energy storage and was connected to the 48 V DC bus through a bidirectional DC converter. An ARM-based breakout module has been used to implement the P&O-MPPT and centralized power-sharing control algorithms.

The controller’s primary function is to regulate and maintain the bus voltage while ensuring continuous and efficient energy delivery, considering both resource availability and load demand. For this purpose, operating modes have been created for the proposed system. The energy flow in the LVDC power bus in operating modes is summarized in Table 2.

**Figure 4:**
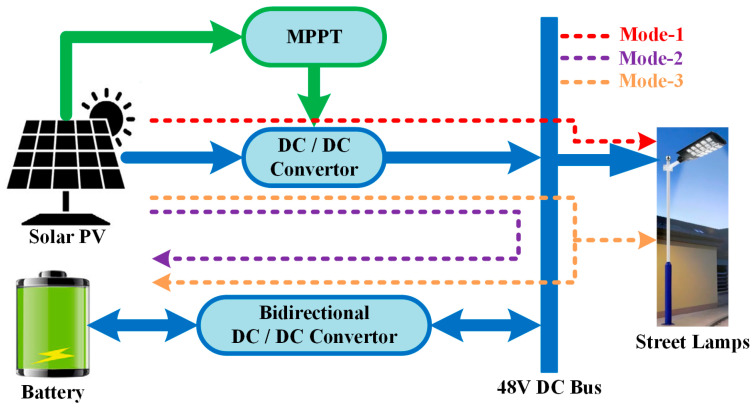
Power flow diagram between system components in the Mode 3 study.

Mode 1 is the operating method in which the solar energy system’s output is assumed to be sufficient to meet the load’s energy demand. The energy flow in this mode is indicated by a dotted red line in Figure 2. In the Mode 2 operating condition, the streetlight does not require energy, and the energy produced by the solar panels is stored in the battery. This is illustrated with a purple dotted line in Figure 2. In contrast, the Mode 3 operating condition is a scenario where the energy demand of the streetlight is less than the amount of energy generated by the solar panel. In this case, while the streetlight’s demand is met by solar energy, the excess amount of energy is used to charge the battery. The brown dotted line in Figure 4 shows the power flow between the system components in Mode 3. The battery converter operates as a voltage converter, reducing the voltage level from 48 V DC to 12 V DC, to charge the battery in Mode 2 and Mode 3 operating conditions.

**Figure 5:**
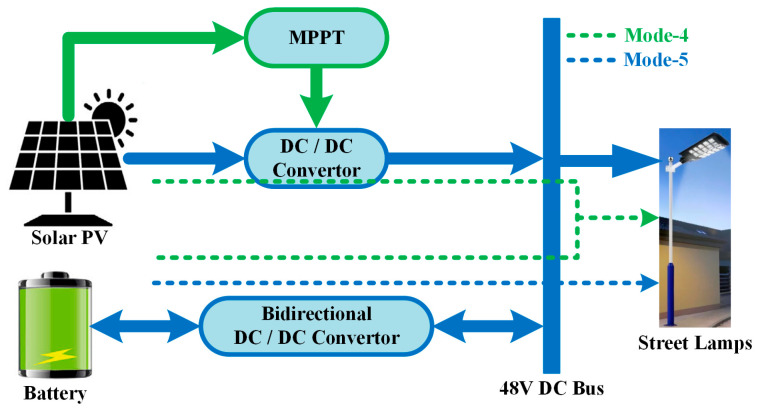
Power flow diagram of the hybrid energy supply for the LED street lamp.

In the Mode 4 study, when energy production from the solar panel is low, the streetlight operates using both solar energy and the battery. Power flow is shown with the green dotted line in Figure 5. The blue dotted line in Figure 5 shows the operation of Mode 5. When energy is not produced from solar energy, energy is transferred from the battery to the streetlight. The bidirectional converter operates in boost mode during Modes 4 and 5 to supply energy to the street lamp. All operating modes ensure that the ARM processor-based control algorithm maintains the bus voltage and meets the streetlight’s demand.

To ensure a continuous supply of energy to the smart streetlight, it is necessary to manage hybrid energy sources, including solar energy and batteries. A power-sharing algorithm was developed using MATLAB/Simulink 2024a platform code blocks. This algorithm operates based on the parameters of the power produced by solar energy (Ppv), the power required by the load (Pload), and the battery’s state of charge (SOCbat). According to the developed algorithm, if Ppv > Pload, the PV power is transferred to the load and the battery. Conversely, if Pload > Ppv, the load is hybrid-fed by the PV power and the battery, depending on the battery’s state of charge (SOC). In conditions where the power generated by the photovoltaic source is greater than the power consumed by the load (Ppv > Pload), the algorithm regulates the bidirectional converter to operate in buck mode by issuing a corresponding control signal. When Pload > Ppv and SoCESS < 25%, the converter switches to boost mode and continues to operate. The voltage reference is set at 50 V, and to regulate the DC bus at this level, the controller activates the boost mode of the bidirectional converter when the photovoltaic (PV) output voltage drops below the predefined threshold. Thus, the converters in the system ensure the continuity of energy transferred to the lamp thanks to the developed control algorithm.

### 2.2. Development of MPPT and Control Algorithm Using Microcontroller

**Figure 6:**
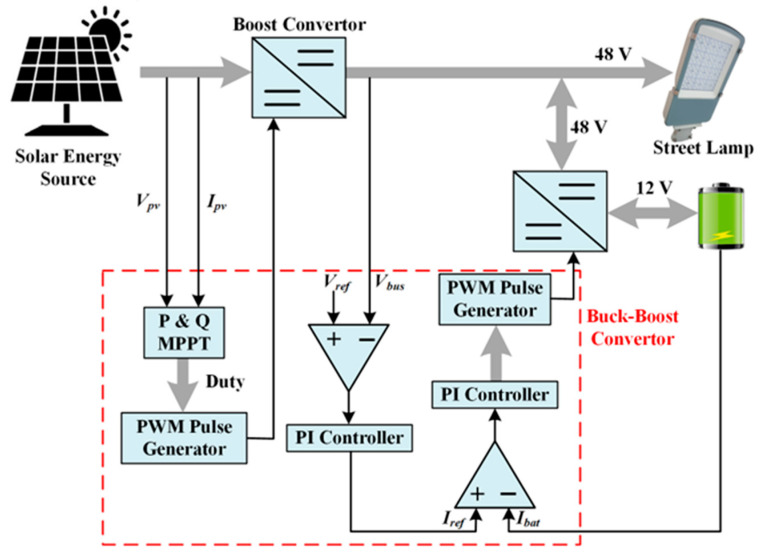
Block schema of street lamp energy bus.

This study focuses on developing and implementing an MPPT algorithm and an integrated control strategy for a LoRaWAN-based street lighting system utilizing an ARM-based microcontroller. The system architecture, including the energy management and control components, is illustrated in Figure 6. The controller is designed to perform two essential functions: (i) real-time MPPT to maximize solar energy extraction, and (ii) coordinated power flow management between the energy sources and storage elements. The P&O-MPPT algorithm processes real-time voltage and current measurements to generate pulse-width modulation (PWM) signals that drive the boost converter for optimal photovoltaic output. Simultaneously, a bidirectional DC-DC converter, regulated based on feedback from the bus voltage and battery current, ensures voltage stability on the energy bus. This converter operates in either buck or boost mode, with switching control achieved using a unified carrier-based PWM generation scheme. A proportional-integral (PI) controller governs the modulation process, ensuring that the bidirectional converter functions exclusively in one mode at a time, thereby enabling seamless and efficient mode transitions.

MPPT Algorithm

**Figure 7:**
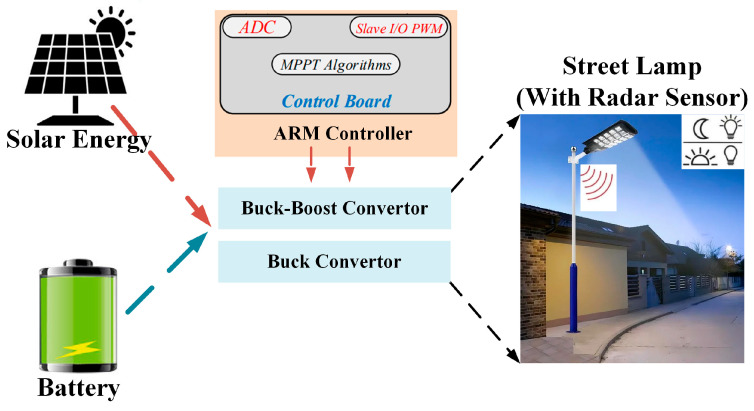
Block diagram of the implementation of the P&O-based MPPT technique.

The MPPT algorithm ensures that solar panels operate at or near their maximum power point under changing conditions such as solar irradiance, temperature, and electric load. The P&O method is one of the MPPT algorithms. The P&O method is widely used in MPPT applications due to its simple structure and high efficiency [29]. In this study, the P&O-MPPT technique was employed to track the Maximum Power Point (MPP). The block diagram of the hardware structure prepared for this purpose is given in Figure 7. The P&O-MPPT technique has been applied to the Buck and Buck-Boost converters using an ARM controller.

**Figure 8:**
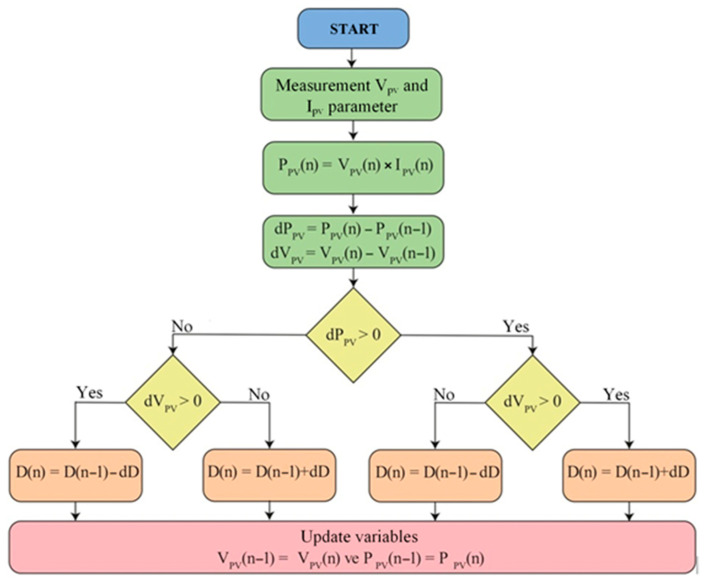
P&O algorithm flowchart.

The proposed method is one of the most widely used and simplest techniques among MPPT methods. The flowchart of the P&O algorithm is presented in Figure 8. In this method, the system observes the change in power by altering the operating point with a specific increase or decrease. If the change causes an increase in power, the same direction is continued; otherwise, the direction is reversed. The algorithm adjusts the voltage iteratively until it finds the value closest to the optimal power point.

**Figure 9:**
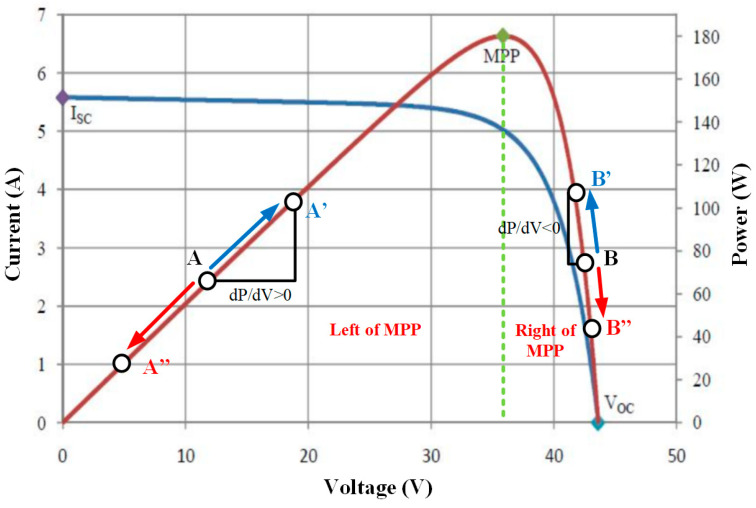
MPPT characteristic curve of the solar panel.

As illustrated in Figure 9, the solar panel’s characteristic curve demonstrates that the power-voltage curve’s slope (dP/dV) is positive on the left side of the MPP, negative on the right side, and zero precisely at the MPP. If the algorithm initially operates at point A and moves to point B due to a perturbation, it will continue perturbing in the same direction as the power output increases, thereby guiding the system toward the MPP. The algorithm functions similarly when approaching the MPP from the right side of the power-voltage curve. Continuously monitoring system performance applies incremental perturbations to the duty cycle, thereby ensuring that the converter operates at or near the maximum power point. The MPPT controller has been developed in MATLAB using the Texas Instruments block set. Using the Simulink block, an ARM-based power sharing control algorithm has been developed in MATLAB. The primary limiting factors of the control algorithm include the power generated by the PV (P_pv), the load power (P_load), and the battery’s state of charge (SOC_bat). As specified in the operating modes, if Ppv > Pload, the PV power is supplied to both the load and the battery. Conversely, if Pload > Ppv, the load is powered by the PV power and the battery, depending on the battery’s state of charge (SOC). The algorithm generates a control signal to operate the bidirectional converter in buck mode when Ppv > Pload. When Pload > Ppv and SoCESS < 25%, the converter is switched to boost mode. The voltage reference is set at 50 V. The controller switches the bi-directional converter’s operating mode to boost mode to maintain the bus voltage when the solar panel output voltage falls below the threshold limit.

This section discusses the simulation results obtained from the FV system model created with the parameters determined on the MATLAB/SIMULINK platform. The system is composed of SunPower SPR-120E panels, each with a power output of 120 W, connected in series. A boost-type DC-DC converter designed for an output power of 120 W was used as the interface between the PV system and the load. The converter is controlled by a control block that enables the implementation of MPPT algorithms, and the duty cycle is dynamically adjusted based on the system’s instantaneous voltage and current data. In this simulation study, a performance comparison was made using three different MPPT algorithms: P&O, Incremental Conductivity (IC), and Particle Swarm Optimization (PSO).

**Figure 10:**
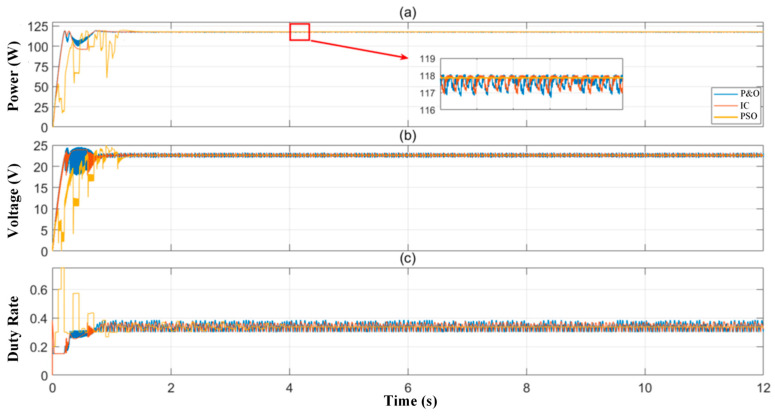
Time variation in (**a**) output power, (**b**) output voltage, and (**c**) duty cycle graphs for P&O, IC, and PSO algorithms under equal irradiance conditions, respectively.

The performance comparison was performed with the simulation conducted under a 120 W constant load, representing standard test conditions where the panel was operating under equal irradiance (1000 W/m^2^). In this case, the P-V curve contains a single maximum power point. The MPPT performances of the P&O, IC, and PSO algorithms under standard test conditions, where all panels operate under equal irradiance (1000 W/m^2^), are presented in Figure 10 in terms of output power, voltage, and duty cycle. As a result of the analyses performed, the output power values of the FV panel were obtained as 117.8 W for the P&O algorithm, 115.7 W for the IC algorithm, and 114.7 W for the PSO algorithm, respectively. Considering the theoretically achievable maximum power value of the panel as 119 W, the efficiencies of these algorithms were calculated as 97.96%, 96.13%, and 96.06%, respectively. Based on the results obtained, the P&O algorithm provided the value closest to the maximum power, achieving the highest efficiency and demonstrating superior performance compared to other methods. Significant differences were observed in terms of monitoring time and steady-state behavior. Specifically, the PSO algorithm exhibited high-amplitude oscillations in the first second, and the transient period was longer compared to other methods.

In the steady state, the voltage response of the FV sequence for the P&O, IC, and PSO algorithms is shown in Figure 10b. After the transition to a steady state following the operation of the P&O, IC, and PSO algorithms, the output voltages of the FV panel were 22.05 V, 22.15 V, and 22.12 V, respectively. All three algorithms exhibited a working voltage close to MPP. The duty cycle change in the converter determined by the MPPT algorithms is shown in Figure 10c. Initially, all algorithms directed the system to increase output power by making a quick task rate adjustment. P&O and IC algorithms showed similar performance in terms of duty cycle change, bringing the system to nominal power level in 0.718 s, while the PSO algorithm exhibited a stable profile in 1.12 s. This also indicates that PSO requires more computation time and parameter tuning.

### 2.3. Hardware and Software Units

**Figure 11:**
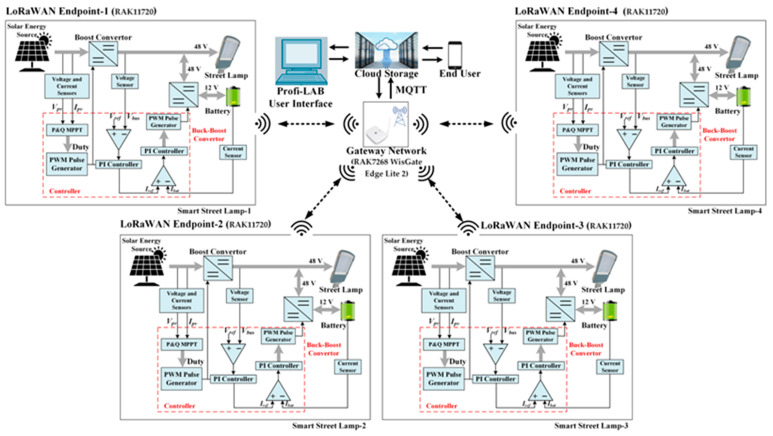
Block scheme of hardware and software units for a smart street lamp.

The system’s overall architecture is illustrated in Figure 11 as a block diagram, showing the interaction between hardware and software units. On the hardware side, the PV panels are connected to the MPPT charge controller, which regulates the charging of the battery storage unit and the direct supply to LED street lamps via the LVDC bus. The LoRaWAN communication module is interfaced with the microcontroller to transmit sensor data and control signals to the central gateway. On the software side, the microcontroller executes the P&O-based MPPT algorithm, processes real-time sensor readings, and manages the switching between operating modes. The gateway forwards the collected data through the MQTT protocol to a cloud platform, where the graphical user interface (GUI) displays system status, energy flow, and performance metrics. This configuration allows for efficient power management, seamless long-range communication, and remote control without internet dependency.

#### 2.3.1. Hardware Units

##### LoRaWAN Communication

Thanks to its advantages, such as low energy consumption and long range, LoRaWAN technology has the potential to be a key technology in IoT-based systems. LoRaWAN is an open communication protocol built upon LoRa modulation, which functions as the physical layer within the OSI model. This technology enables long-range wireless communication, with coverage extending up to 15–20 km in rural areas and 2–8 km in urban environments [46].

The basic components of the LoRaWAN network architecture are end nodes, gateways, a Network server, and an Application server. End nodes represent edge devices or sensors. It transmits the data obtained from the system to the gateway or transfers the data from the gateway to the system. Gateway collects or distributes incoming data from several end nodes. Gateways continuously monitor all frequency channels and relay the received data to the LoRaWAN network server using a TCP/IP connection and a packet forwarder. The network server aggregates data transmitted from multiple gateways and forwards it to the application server. The application server is responsible for processing and/or visualizing the consolidated data.

Low-power wide-area networks can transmit data at a frequency of 915 MHz [34]. The transmission of encoded data at different frequencies makes this technology more secure than other technologies. Since LoRaWAN uses low-frequency signals, they can pass through various obstacles or barriers that could cause problems for transmitting long-wavelength signals. This advantage ultimately leads to the transmission of long-wavelength signals over greater distances. Since the 915 MHz frequency used by LoRaWAN is vacant, this technology’s devices are not affected by noise. Using LoRaWAN for communication sometimes requires additional hardware (antennas and nodes). LoRaWAN does not require authentication because it operates in the 915 MHz band and does not require an additional license [35]. The long-range data transmission capability makes it suitable for various projects, such as smart city initiatives, that utilize different types of sensors to gather information.

The RAK11720 Breakout Board (Endpoint) monitors and controls the streetlights. The RAK11720 Breakout Board wirelessly transfers data received from the streetlights to the gateway or sends control parameters via the gateway to the Breakout Board. RAK7268 WisGate Edge Lite 2 brand gateway was preferred as the receiver of the breakout modules. The gateway is a fully integrated 8-channel indoor device that operates on the LoRaWAN protocol and features built-in Ethernet connectivity, enabling a simplified and reliable installation process.

##### LED Lamp and Sensor Design

**Figure 12:**
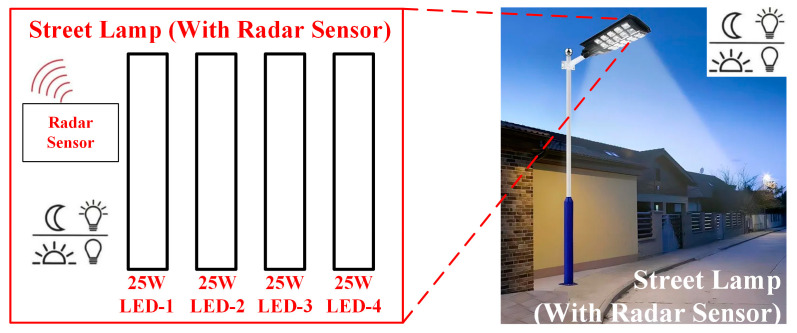
Designed a street lamp model.

The street lamp designed LED street lamp model comprises four 25 W power strip LEDs. The visual of the created model is provided in Figure 12. Each strip of LEDs is controlled individually. The radar sensor detects human movement with high-frequency electromagnetic waves at a frequency of 5.8 GHz. Thanks to the detection network of the radar sensor placed on the street lamp, when no living beings move in the environment for a specific period, the lamp’s brightness is automatically reduced to 25%. When motion is detected in the environment, the lamp brightness increases to 100%. Thus, the application ensures optimal energy efficiency. Additionally, a solid-state relay has been used to activate and deactivate the streetlight. Since this relay allows for ON/OFF operation with 3.3 V or 5 V DC voltage, it has been used directly in the study.

##### Current and Voltage Sensors

**Figure 13:**
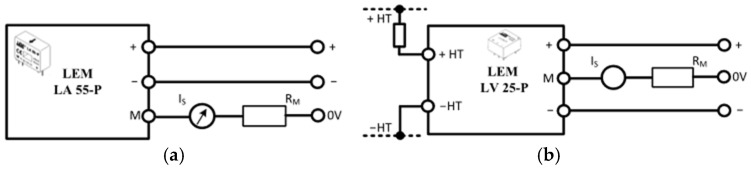
Current and voltage sensors, (**a**) LA-55 sensor connection, (**b**) LV-25P sensor connection.

A current-voltage reading circuit has been designed to analyze the phase voltages and currents at the circuit outputs using the LA-55 current sensor and LV-25P voltage sensors. Both sensors operate as closed-loop Hall-Effect transducers. The LA-55 sensor measures currents up to 50 A by passing them through the transmission line. In Figure 13a, the pin outputs of the LA-55 current sensor are shown. It operates with a ±15 V symmetric power supply and provides the measured line current as AC at a 1:1000 conversion ratio from the M (measurement) output. The measured current output is amplified in proportion to the number of turns of the transmission line passing through the gap in the middle of the sensor. The LV-25 P voltage sensor provides the measured voltage at the M measurement point by relating it to the current passing through an external R resistor. For the sensor with a conversion ratio of 2500:1000, the recommended value for the R1 resistor (Figure 13b) to be used for measurement is 25 kΩ.

#### 2.3.2. Software Units

##### MQTT Communication Protocol

MQTT is a protocol specifically designed for IoT applications, characterized by low data volume and energy efficiency. This allows devices to operate for extended periods with low energy consumption. MQTT works through a central broker, so sensors or devices publish data, and users or other devices subscribe to this data. Thus, it reduces the complexity of the devices in the system. This protocol ensures reliable data transmission even under low bandwidth conditions. This provides good compatibility with long-range and low-speed networks, such as LoRaWAN.

##### SCADA Interface

The user interface was created using the Profi-LAB Expert program, a human–machine interface software. This program is a system software designed for the comprehensive and detailed examination, monitoring, and control of industrial processes. It consists of components related to software and hardware protocols. This system allows users to access, collect, analyze, and report real-time information during production. The SCADA interface is created using code blocks in the Profi-Lab program. Data transfer occurs every 1 ms, and the data is stored in a table. This data in the table is processed and presented to users through the components within the interface.

## 3. Experimental Results

**Figure 14:**
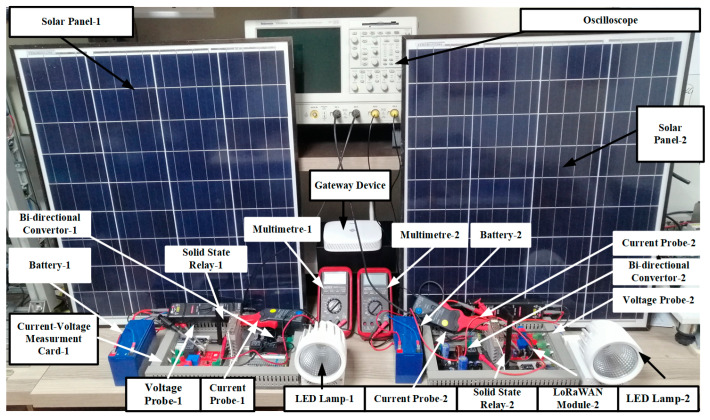
General view of the experimental set.

Figure 14 shows the experimental setup of the smart street lighting. It is worth remarking that the experimental results were obtained by using four solar panels. Two of these panels are shown in Figure 14 to demonstrate the setup details. A LoRaWAN-based monitoring and control unit is provided for each lamp group. Accordingly, each unit has a battery, solar panel, current-voltage reading card, LED streetlight, timer, LoRaWAN communication card, and ON-OFF control switch. The data obtained from each unit is collected via the Gateway and transferred to the user interface through the cloud.

Figure 13 depicts the output profiles of the PV system in terms of power, voltage, and current. The MPPT algorithm continuously monitors system conditions to accurately identify and maintain operation at the MPP, thereby maximizing energy extraction. The system is subjected to frequently varying environmental conditions to validate the dynamic tracking performance of the proposed MPPT scheme. These variations are experimentally introduced by altering the temperature and solar irradiance levels through partial shading of the PV panel under standard illumination. The technical properties of the solar panel used in the setup are a 680 × 880 × 25 mm panel size, a maximum power voltage of 20.84 V, a maximum power current of 5.47 A, an open circuit voltage is 24.72 V, and a Short Circuit current of 5.77 A. Test operations are implemented in the garden of Gazi University Campus.

**Figure 15:**
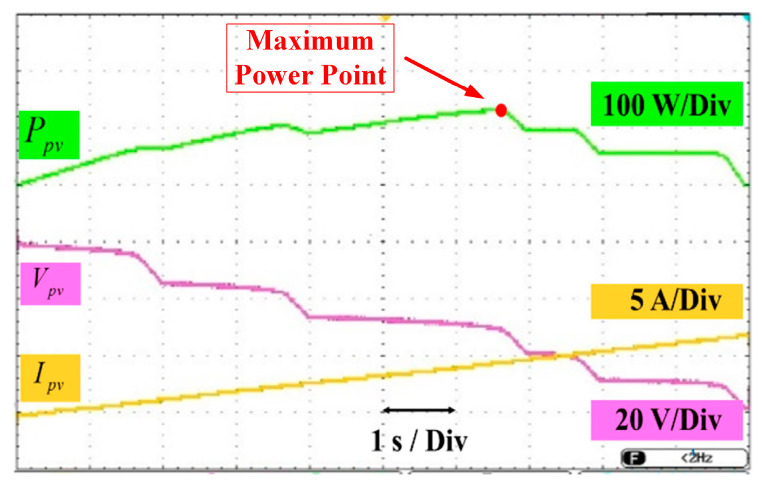
The P-V and V-I Curve of The Solar Panel (P-V and V-I Curve at the Maximum Power Point of The Solar Panel).

The P&O method is adopted in this study owing to its algorithmic simplicity, real-time applicability, and widespread use in PV systems. The proposed algorithm is developed to dynamically monitor input variations and adapt the converter’s duty cycle accordingly, facilitating continuous operation at the MPP to optimize overall power extraction. Figure 15 presents the load current waveforms, load voltage, and load power under varying load conditions. The results indicate that the controller effectively adjusts the converter’s operating point to regulate the DC bus voltage, maintaining its stability despite fluctuations in the load profile.

**Figure 16:**
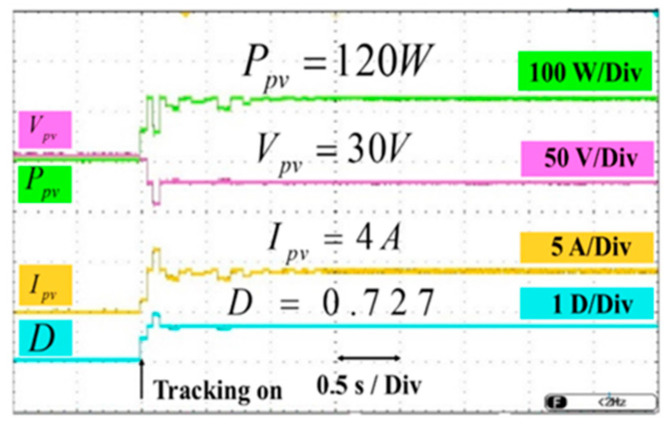
Experimental results for achieving maximum power using the P&Q algorithm.

The proposed MPPT approach has a maximum tracking power of 120 W under standard test conditions using a constant irradiance of 1000 W/m^2^. As shown in Figure 16, this algorithm has the maximum efficiency of 97.96% among the examined MPPT approaches, as calculated using Equation (1). The P_pv-steadystate_ parameter represents the power value of the solar panel in a stable operating condition, and P_max-available_ represents the maximum power to be drawn from the solar panel.

(1) $$Efficiency=\frac{P_{pv\text{-}SteadyState}}{P_{max\text{-}Available}}\times 100$$

**Figure 17:**
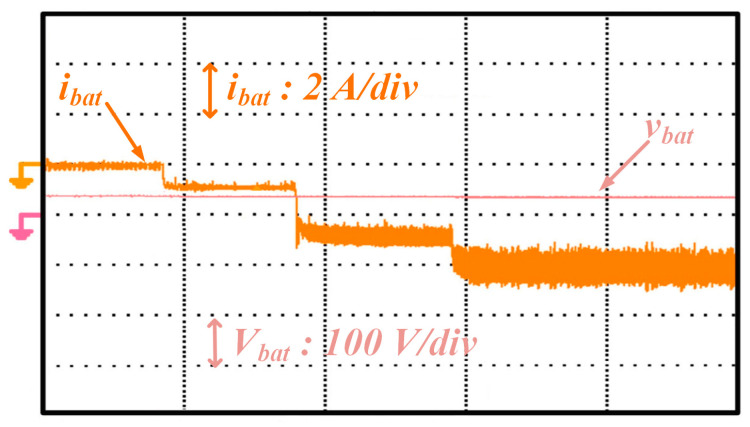
Experimental results when only the battery powers the street lamp.

Figure 16 illustrates experimental results for obtaining maximum power using the P&Q algorithm. The experimental results obtained when the street lamp is powered solely by a battery are shown in Figure 17. The figure displays the battery voltage (Vbat) and battery current (Ibat). This study is for the case where the battery and solar panel circuits are absent. The battery voltage has been stabilized at 48 V DC using a PI-controlled boost converter. The battery’s discharge current has been set to 2.1 A for a maximum power of 100 W. The street lamp was tested by activating different LED groups for 25 W, 50 W, 75 W, and 100 W power levels.

**Figure 18:**
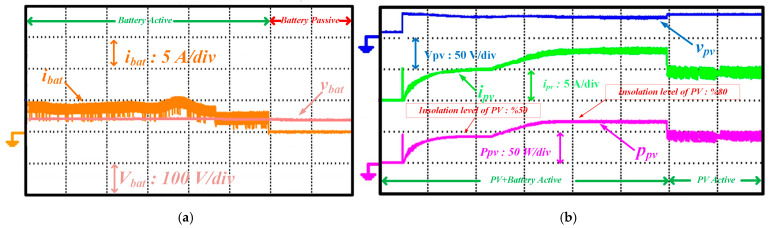
Waveform of different quantities (**a**) 50% sunlight from solar energy, (**b**) the sunlight level is 80% while the PV converter changes its operation from MPP to non-MPP.

To demonstrate the transition of energy obtained from the solar panel from MPP to non-MPP, the experimental results are shown in Figure 18a,b. When there is 50% sunlight from solar energy, it is activated to obtain a 48 V DC bus voltage. When the solar panel is in operation, the battery is also connected. As seen in Figure 18b, when the sunlight level is 80%, the power value obtained from solar energy is greater than the power value provided by the battery at that moment, so the battery energy is disconnected, and the lamp continues to be powered by the solar panel.

**Figure 19:**
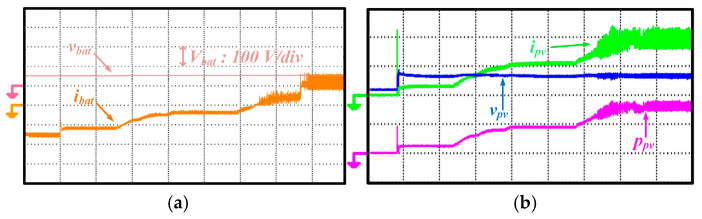
Waveform of different quantities (**a**) battery parameters (**b**) solar parameters while the battery converter changes its operation from discharging to charging.

The transition of the battery converter from discharge mode to charge mode while operating in discharge mode is shown in Figure 19a,b. While the battery provides the street lamp’s energy supply, solar energy is initially out of the circuit. Later, the PV starts operating at a 10% sunlight level, and after a specific period, the sunlight level rises to 100%. This change in PV energy causes the battery inverter to switch from discharge to charge mode. Thanks to this transition, the powering of the streetlight varies according to the power potential of the energy sources, ensuring that the streetlight is continuously powered.

The results of these experiments show that the energy management of the hybrid energy source consisting of solar energy and batteries has been successfully achieved thanks to the developed algorithm. This system has been applied separately to four LED lamps located 400 m away. A LoRaWAN-based application has been developed via a computer for remote monitoring and control of system parameters related to energy management.

LoRaWAN technologies are essential in IoT scenarios due to their wide radio ranges, use of unlicensed frequency bands, and low energy consumption. Among these technologies, long-range WAN (LoRaWAN) is widely used in smart city monitoring, precision agriculture, and environmental monitoring, transmitting over several kilometers with data rates of up to 5.5 kb/s. In this study, a LoRaWAN-based smart campus street lighting system was implemented. The system includes monitoring and controlling four LED lamps, each located 400 m apart, over an open area of approximately 1.2 km, without the need for the internet. The LoRaWAN communication method, which can be developed to provide two-way communication, has been preferred as a communication technology. The data packet size in the system is set to 10 bytes, and this small data size contributes to energy savings. The user can monitor or control each streetlight or group of streetlights integrated into the system through a secure and user-friendly application interface that is accessible remotely. The LED lamp’s energy line uses a hybrid energy source consisting of a solar panel and battery system. Thanks to the created optimization workflow, maximum energy efficiency and flexibility are achieved in the management of streetlights. Each lamp can be turned on/off remotely, the lighting level of the lamps can be managed, and the monitoring and control of lamp parameters can be performed digitally and graphically through the user interface.

**Figure 20:**
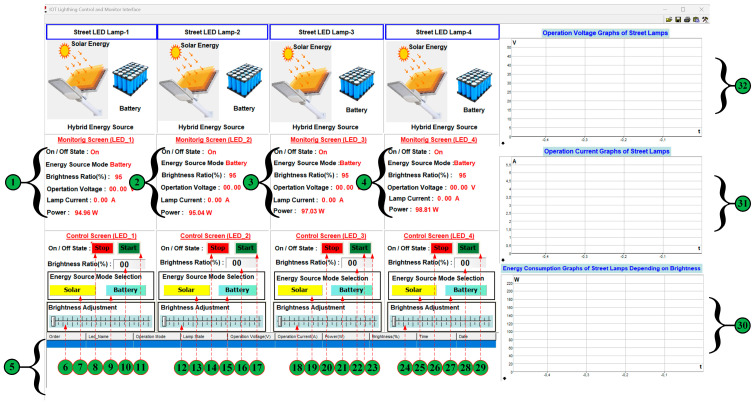
Screenshot of the user interface designed in the Profi-LAB Expert editor program.

The picture of the user interface is shown in Figure 20. This interface is designed in the Profi-LAB Expert editor program. The monitoring and control of system parameters are carried out through this interface using code blocks. The status information, energy source mode, brightness level, voltage, current, and power parameters of four streetlights are automatically transmitted to the user interface via the LoRaWAN communication network, and these data are displayed numerically to the user. In the control process of four streetlights through the user interface, the on/off control of the lamps, brightness level, energy source mode selection, and brightness adjustment control can be performed. The current, voltage, and brightness-dependent energy consumption of each street lamp is displayed graphically on the user interface, and the monitoring and control parameter data for these lamps are transmitted to the user interface via a cloud-based LoRaWAN communication network. These data are shown in tabular form in the area marked with number 5 in Figure 20. The detailed explanation of each street light’s monitoring and control parameters in the user interface shown in Figure 19 is numbered and provided in Table 3.

**Figure 21:**
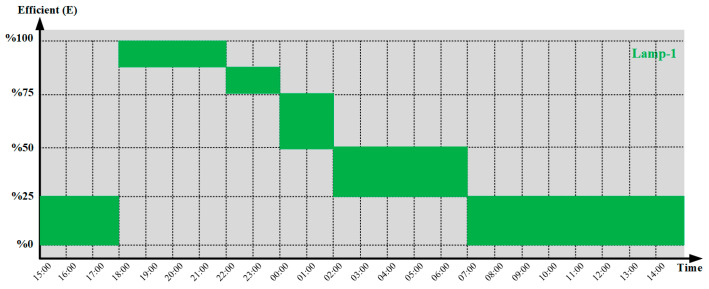
Daily operating curve of the street light (for LED_1).

Figure 21 shows a performance graph using the daily operating duration data for each street lamp, indicated by the number 5. For example, the performance graph of the first street lamp is shown in Figure 21. Accordingly, it is observed from the performance curve that the street light operates at 100% brightness between 18:00 and 22:00, and at different brightness levels between 22:00 and 07:00.

**Figure 22:**
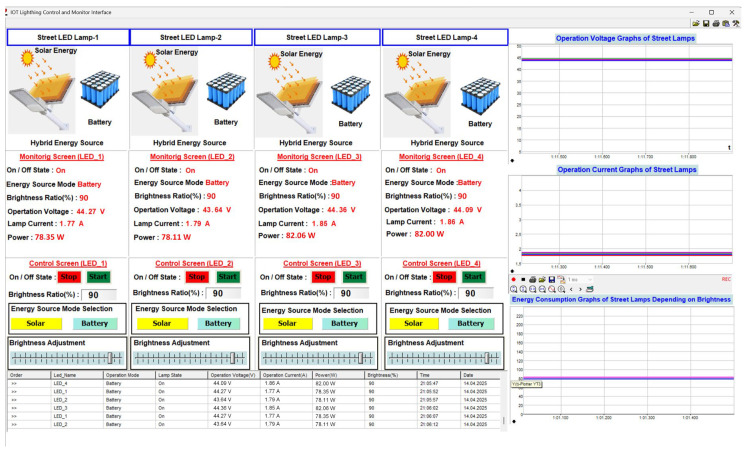
The user interface screenshot when the brightness level of each street lamp is 90%.

The monitoring and control parameters for each street lamp are displayed on the user interface, as shown in Figure 22. The data related to the monitoring parameters are transferred to the table on the user interface with a sampling time of 100 us and recorded in an Excel table. The working mode of each street lamp, including the status of whether the lamp is on or off, the current drawn from the source, the operating current, power, brightness, and recorded time information, is graphically displayed to the user. The brightness level of each street lamp is 90%, and while operating in battery mode, the current drawn from the source is measured as 1.77 A, 1.79 A, 1.85 A, and 1.86 A, respectively, the operating voltage as 44.27 V, 43.64 V, 44.36 V, and 44.09 V, respectively, and the amount of energy consumed as 78.35 W, 78.11 W, 82.06 W, and 82.00 W, respectively. These values are displayed both numerically and graphically on the user interface. When the brightness level of each street lamp is changed through the user interface, this change is instantly reflected on the street lamp and subsequently in real-time on the operating parameters of the lamps displayed on the user interface. The selection process for the energy source of the streetlight can also be performed through the user interface. This selection process can use solar energy, batteries, or hybrid systems. As seen in the current graph in Figure 22, the obtained data is recorded in real-time, just like in the other graphs. Graphical operations such as horizontal or vertical zooming, control of the graph transfer speed, etc., can be performed on recorded or real-time data.

**Figure 23:**
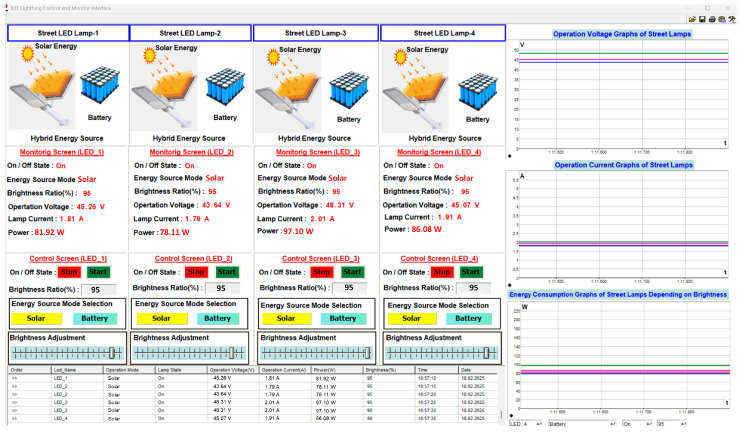
The user interface screenshot when the brightness ratio of all lamps is 95%.

In the selection process of the energy source, when solar energy and brightness ratio are selected at 95%, the user interface appears as shown in Figure 23. The change in brightness ratio directly affects the voltage level applied to the street light, the current drawn by the lamp, and the amount of energy consumed. This change can be observed numerically and graphically from the monitoring parameters in Figure 23. The brightness level of each street lamp is 90%, and while operating in solar energy mode, the current drawn from the source was measured as 1.81 A, 1.79 A, 2.01 A, and 1.91 A, respectively, the operating voltage as 45.26 V, 43.64 V, 48.31 V, and 45.07 V, respectively, and the energy consumption as 81.92 W, 78.11 W, 97.10 W, and 86.08 W, respectively.

**Figure 24:**
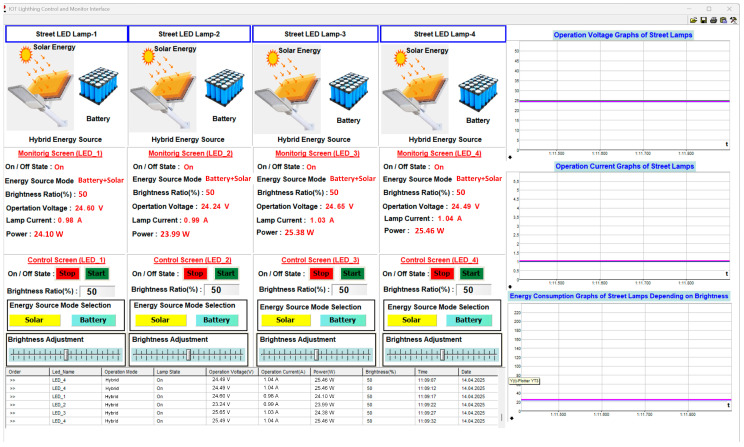
The user interface screenshot when the brightness ratio of all lamps is 50%.

The process of selecting the energy source, both solar energy and battery, and when the brightness ratio is set to 50% (hybrid mode), the user interface appears as shown in Figure 24. The brightness level of each street lamp is set to 50%, and while operating in solar energy mode, the current drawn from the source is measured as 0.98 A, 0.99 A, 1.03 A, and 1.04 A, respectively, the operating voltage as 24.60 V, 24.24 V, 24.65 V, and 24.49 V, respectively, and the energy consumption as 24.10 W, 23.99 W, 25.38 W, and 25.46 W, respectively. Thanks to the energy management system that operates according to the potential of the energy sources, hybrid operation is achieved. Thus, the energy produced from solar power can charge the battery and meet the load’s demand.

**Figure 25:**
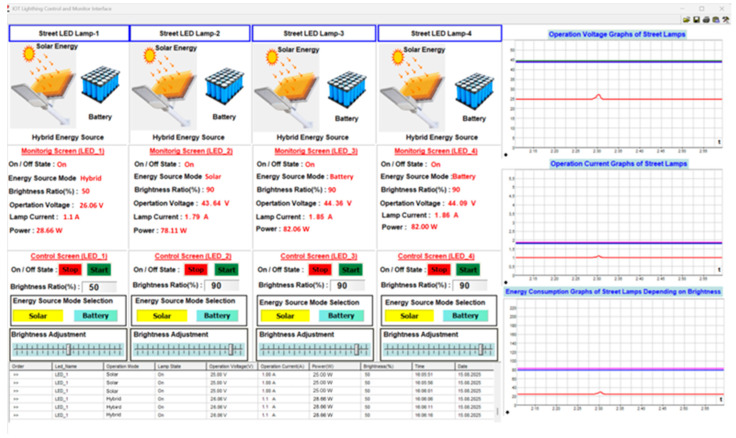
The user interface screenshot when the transition from solar energy to a hybrid energy source (for LED_1).

When the brightness ratio for LED_1 is set to 50% and the power source switches to battery when solar energy is unavailable, the source transition user interface appears as shown in Figure 25. The energy source transition was only inspected for LED_1, while the energy source for LED_2 was set to solar energy, and the energy sources for LED_3 and LED_4 lamps were set to batteries.

The brightness ratio for the LED_1 lamp is selected as 50% and while operating in hybrid energy mode, the current drawn from the source is measured as 1.1 A, the operating voltage as 26.06 V, and the energy consumption as 28.66 W. The brightness level of LED_2, LED_3 ve LED_4 street lamp is set to 90%, and while operating in solar or battery energy mode, the current drawn from the source is measured as 1.79 A, 1.85 A, and 1.86 A, respectively, the operating voltage as 43.64 V, 44.36 V, and 44.09 V, respectively, and the energy consumption as 78.11 W, 82.06 W, and 82.00 W, respectively. Thanks to the energy management system that operates according to the potential of the energy sources.

**Figure 26:**
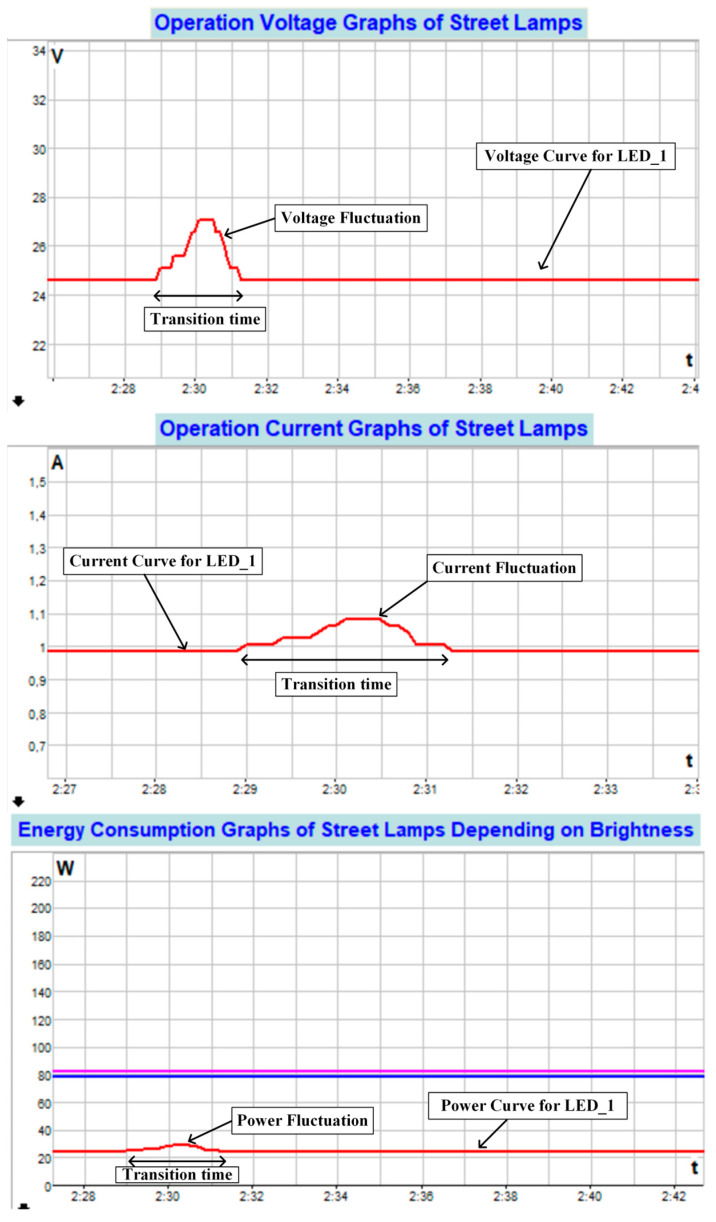
Screenshot of the user interface for current, voltage, and power graphs when transitioning from solar energy to a hybrid energy source (for LED_1).

The screenshot of the user interface for current, voltage, and power graphs when transitioning from solar energy to a hybrid energy source is shown in Figure 26. As can be seen from the graphs in Figure 26, during the transition from solar energy to a hybrid energy source, there was a voltage fluctuation of approximately 2 V for about 2 s, resulting in a current fluctuation of approximately 0.1 A. When the transition period is complete, the system continues to operate stably as before. In this case, it is observed that the dynamic response of the proposed control and management algorithms in the transition from solar energy to a hybrid energy source for the LED_1 street lamp is high.

The user can remotely manage the outdoor lighting infrastructure by adjusting optional dimming levels to optimize energy consumption and receive real-time feedback from streetlights. This capability not only reduces operational and maintenance costs through precise scheduling of on-site service tasks but also enhances the reliability and energy efficiency of the environmental lighting system. This application study aims to limit energy consumption, minimize light pollution and CO_2_ emissions, provide optional lighting, ensure sustainability without compromising comfort and safety, significantly reduce maintenance costs, and lower operating expenses.

Types of faults, such as energy line problems, solar panel malfunctions, battery failures, lamp issues, and sensor faults, are monitored in real-time through a cloud-based user interface. Real-time energy consumption reporting, with graphical data for each street lamp, ensures the optimization of the connected lighting infrastructure. Thanks to rich visualization data, the most efficient energy optimization has been achieved through maintenance planning.

## 4. Discussion

This section presents a critical analysis of the experimental results. First, it can be seen in Figure 9 that the MPPT efficiency of 97.96% obtained by the P&O algorithm in this system shows competitive performance compared to the simulation study. An ARM microcontroller-based control system was used to maintain voltage and current stability under variable loads. This high-resolution processor ensures system stability through the energy management algorithm by enabling the DC-DC converters in each lamp structure to operate at a switching frequency of 10 kHz.

There are many works in the literature regarding the design and development of smart street lights. The proposed study is compared in Table 1 with the four different existing studies introduced in [54,55,56,57]. These studies were conducted based on the proposed energy type, monitoring and control feature, energy management feature, and control and communication type.

As shown in Table 4, [54] proposes a Wi-Fi-based lighting monitoring and control system for energy efficiency. The control method in [51] is not robust to parameter variations, while the other methods are robust. The dynamic responses of the control methods can be specified as the key performance indicator. The dynamic responses of the proposed method are investigated under variations in all system variables, and satisfactory results are obtained as discussed in the experimental results. Since the effect of the variations in some system variables is not discussed in [55,56,57], the performance of the control methods in these papers is uncertain.

The proposed method was tested at a higher power value compared to [54,55,56,57], as shown in Table 4. The hybrid energy source (solar and battery) is important in practical applications due to its simplicity and low system cost. The proposed method was implemented using a hybrid (solar and battery) energy source. Additionally, thanks to the MPPT algorithm, maximum energy storage from solar power to the battery can be achieved.

The lamp power in the smart street lighting system is higher than the methods in [54,55,56,57], as shown in Table 4. Additionally, the fact that the system’s energy source consists of solar energy, batteries, and hybrid energy independent of the grid, and that this energy is managed, highlights the ease of implementation of the proposed method. In conclusion, the proposed study offers currentness, practical application, and system cost advantages. In addition, this application provides free communication and a high energy saving potential in rural areas without electricity or internet access, thanks to the ability to monitor without internet and motion detection-based lighting.

Table 5 gives a comparison of the characteristics of this system with previous studies using wireless communication technologies to highlight the advantages of LoRaWAN in terms of range and energy efficiency. Wireless communication technologies are integral to the functionality of IoT systems, enabling seamless data transfer between sensor devices, IoT gateways, and other system components. These technologies vary based on factors such as communication range, bandwidth, and power efficiency. In PV and battery energy systems, ensuring reliable communication is particularly important, especially in remote locations where solar plants are often situated. Therefore, selecting technologies that support continuous, real-time, and energy-efficient data transfer is crucial for monitoring performance, managing energy generation, and enabling remote maintenance.

Short-range technologies are well-suited for localized monitoring and control, ensuring the efficient operation and integration of PV components within smart energy networks. Wi-Fi, for instance, is commonly used for monitoring energy production and system performance, allowing remote access and control. However, its high energy consumption makes it less ideal for energy-sensitive applications like PV systems, where energy efficiency is paramount. Bluetooth Low Energy (BLE) is a cost-effective communication technology with a range of up to 30 m, making it ideal for small-scale IoT applications in hybrid energy systems. BLE is particularly useful in energy management systems for monitoring and controlling hybrid energy systems in smart homes and offices, enabling optimized energy consumption through enhanced communication between devices. Zigbee, designed for low-power applications with a communication range of up to 100 m, is widely used in PV systems. It supports smart grid integration, energy monitoring, and home automation, optimizing energy usage by enabling seamless communication between PV components such as inverters, sensors, and controllers. LoRaWAN technology is crucial for allowing long-range, energy-efficient communication in IoT systems. The technology is particularly valuable for remote monitoring and managing hybrid energy systems, especially on a large scale. LoRaWAN offers long-range communication (over 10 km in rural areas) with low energy consumption, making it ideal for smart grids. In hybrid energy systems, LoRaWAN is used for energy management and monitoring over large distances, particularly in remote areas. Table 5 presents a comparison of wireless communication Technologies [58].

Limitations of the study

This study has certain limitations in terms of the materials used, cyberattacks, implementation, and economic aspects. These limitations can affect the generalizability and economic feasibility of the results. In large-scale applications, the increasing number of connections and hardware complexity have the potential to increase maintenance and production costs. Below are the main limitations of the study and suggestions for future research:

The Impact of Cyberattacks on Efficiency

Cybersecurity is an important factor that can directly affect productivity values. Like any wireless communication system, LoRaWAN can have vulnerabilities that attackers can exploit. Understanding these risks is crucial for protecting LoRaWAN networks and implementing robust security measures to ensure the integrity and confidentiality of data transmitted over these networks. Network attacks can target vulnerabilities in the LoRaWAN network infrastructure. These attacks can include unauthorized access to network resources, unauthorized network reconfiguration, or the addition of malicious network traffic.

Limitations in the Application Section

Network Coverage: While it offers advantages in rural areas, signal interference, high-rise buildings, or electromagnetic interference can lead to coverage issues in dense urban areas. This situation can be resolved by performing mesh operations between communication structures or by placing gateways in suitable locations to eliminate signal loss and data transfer issues.

Hardware compatibility: Network incompatibilities can occur if devices from different manufacturers are not compliant with LoRaWAN standards. This situation can be resolved by choosing devices with the same technical specifications or products from the same brand.

Unlicensed frequency use: LoRaWAN operates in unlicensed bands, which can lead to signal interference and noise in some areas. This problem can be eliminated by paying attention to belt selection.

This study only considered material costs in its economic evaluations. Production, installation, and operating costs were assumed to be constant and excluded from the evaluation. However, a comprehensive system-level economic analysis is needed to better understand long-term cost-effectiveness.

Considering the limitations of the study, it is suggested to focus on the following topics in the future:

- The feasibility of this proposed approach could be evaluated in larger-scale smart city projects;
- Integration with artificial intelligence (AI) and machine learning (ML) algorithms can be achieved;
- The development and optimization of multi-sensor systems can be ensured;
- Research can be conducted in the area of improving channel modeling and network configurations;
- Prevent LED failures and LED driver failures by detecting voltage fluctuations in used smart campus or smart city street lighting;
- Implementing different security mechanisms over smart lighting protocols.

## 5. Conclusions

This research proposes an efficient off-grid street lighting solution based on P&O-MPPT using LoRaWAN communication without internet access. This solution is scaled to a four-street lamp setup. The LoRaWAN communication method was chosen for this study because it offers long range, low energy consumption, low installation cost, and high data capacity compared to other methods. The primary objective of this study is to present a design for a street lighting system based on LEDs, which is hybrid-powered by solar energy and batteries, thereby making it independent of the grid. Thanks to the developed energy management algorithm, the battery is charged to its maximum capacity with solar energy during the day, and this stored energy is used in street lighting in a controlled manner at night. The MPPT algorithm was used to achieve maximum solar energy production. It focuses on reducing energy consumption during times of low demand, managing energy according to the potential of energy sources, and enhancing system reliability by enabling monitoring and control of the entire system through the LoRaWAN communication method.

This study’s first distinction from other studies in the literature is its commitment to energy efficiency and reduced energy consumption. Its second distinction is the creation of an energy harvesting subsystem powered by solar and battery power. Its third distinction is its provision of a cost-effective and environmentally sustainable solution that meets the needs of both urban and rural areas. Its final contribution to the literature is using IoT-based control systems to achieve energy savings and improve lighting quality.

This system reduces operational and maintenance costs and supports environmental sustainability goals by minimizing CO_2_ emissions and light pollution. Furthermore, it aligns with the United Nations Sustainable Development Goals (SDGs) on clean energy and resilient infrastructure. Future work will enhance the system’s intelligence by integrating machine learning algorithms for predictive maintenance and adaptive lighting control, expanding sensor networks for environmental data collection, improving cybersecurity through blockchain-based solutions, and investigating energy sharing among nodes to optimize distributed resources. Additionally, the system’s scalability for larger urban and rural applications and its potential integration into smart grid infrastructures offer promising avenues for continued research and real-world implementation.

## Figures and Tables

**Table 1:** Comparison of wireless communication methods for IOT.

	BLE	Wi-Fi	Z-Wave	Zigbee	LTE-M	NB-IOT	Sigfox	LoRaWAN	GPRS
Average range	50 m	1000 m+	30 m	100 m	2–5 km	20 km+	10 km+	10 km+	35 km+
Network Type	PAN	LAN	PAN	PAN	LPWAN	LPWAN	LPWAN	LPWAN	WAN
Bandwidth	1 MHz	20 MHz	-	3 MHz	-	180 KHz	100 KHz	125 kHz	-
Speed	1 Mbps	150 Mbps	100 Mbps	250 Kbps	1 Mbps	250 Kbps	100 bps	0.3–50 kbps	85 kbps
Package Size	47 Bytes	2304 Bytes	64 Bytes	127 Bytes	-	-	12 Bytes	256 Bytes	-
Standart	IEEE 802.15.1	IEEE 802.11	Z-Wave Alliance	IEEE 802.15.4	3 GPP	3 GPP	IEEE 802.15.1	IEEE 802.15.g	ETSI
Energy Consumption	10 mW (+year)	High (week)	Very Low (+year)	Very Low (+year)	Low (month)	Low (month)	Low (month	Very Low (+year)	High (week)
Installation cost	One time	One time	One time	One time only	Continuous	Continuous	Continuous	One time	-
Module cost	<5$	<5$	<5$	8–15$	8–20$	8–20$	<5$	8–15$	-
Topology	P2P, Star, Mesh	Star, Star, Mesh	Mesh	Mesh	Star	Star	Star	Star	Star

**Table 2:** Summary of energy flow in LVDC power bus operating modes.

Modes (1–5)	Energy Flow	Explanation
Mode-1	There is a flow of energy from the solar panel to the streetlight.	The street lamp receives its energy from a solar panel. The system automatically decides on resource selection based on the amount of energy in energy sources, thanks to the developed energy management.
Mode-2	There is a flow of energy from the solar panel to the battery.	The streetlight does not require energy, and the energy produced by the solar panels is stored in the battery. (reducing the voltage level from 48 V DC to 12 V DC)
Mode-3	While the streetlight’s demand is met by solar energy, the excess amount of energy is used to charge the battery.	The battery converter operates as a voltage converter, reducing the voltage level from 48 V DC to 12 V DC, to charge the battery.
Mode-4	There is a flow of energy from both the solar and the battery to the streetlight.	When energy production from the solar panel is low, the streetlight operates using both solar energy and the battery.
Mode-5	There is a flow of power from the battery to the streetlight.	When energy is not produced from solar energy, energy is transferred from the battery to the streetlight.

**Table 3:** Explanation of monitoring and control parameters for each street light on the user interface.

The Number on the Interface	Explanation
1, 2, 3, and 4 numbers	It represents the monitored operational parameters for each streetlight. The information on whether the streetlight is working or not, the type of energy source (solar panel or battery), the lamp’s brightness level, operating voltage, current drawn from the source, and the energy consumption of the lamp are monitored in real-time through a LoRaWAN-based cloud system.
6, 7, 8, 9, 10, and 11 numbers 12, 13, 14, 15, 16, and 17 numbers 18, 19, 20, 21, 22, and 23 numbers 18, 19, 20, 21, 22, and 23 numbers 24, 25, 26, 27, 28, and 29 numbers	It expresses the operational parameters related to the control of each streetlight. The streetlight’s On/Off control, lamp brightness adjustment, and energy source selection (solar panel/battery) are controlled in real-time via a LoRaWAN-based cloud system.
5 number	The operating parameters for each street light are displayed in a table.
30 number	The energy consumption of each street light is being monitored graphically.
31 number	The current value consumed by each street lamp is monitored graphically.
32 number	The voltage value consumed by each streetlight is monitored graphically.

**Table 4:** Comparison of Smart Street Lighting in the Literature.

Ref.	Year	Energy	Monitoring and Control	Energy Management	Control Method	Communication	Lamp Power
[51]	2021	Solar and battery	Control	Yes	Dimmer Control, Battery Management System, and MPPT	Wi-Fi	20 W
[52]	2024	Grid	Monitoring or Control	Yes	Dimmer Control Motion Control	ZigBee	70 W
[53]	2024	Solar and battery	Monitoring or Control	Yes	Motion and Light Intensity Control	No	50 W
[54]	2021	Grid	Monitoring or Control	No	Motion, Current, and Voltage Control	LoRaWAN	70 W
Proposed	2025	Solar, battery and hybrid	Monitoring or Control	Yes	Dimmer Control Motion Control Battery management system and MPPT	LoRaWAN	120 W

**Table 5:** Comparison of wireless communication technologies in PV and battery systems [58].

Technology	Range	Energy Consumption	Applications in PV and Battery Energy Systems
Wi-Fi	≤100 m	High	Monitoring and remote control of energy production and system performance. Less suitable for PV systems.
Bluetooth Low Energy (BLE)	≤30 m	Low	Small-scale PV systems in smart homes and offices.
Zigbee	≤300 m	Very Low	Smart grid integration, energy monitoring, and enabling efficient communication between PV components
LoRaWAN	≤10 km	Very Low	Monitoring and energy management in large-scale and remote PV installations.

## Data Availability

The data presented in this study are available on request from the corresponding author. The data are not publicly available due to privacy.

## Abbreviations

The following abbreviations are used in this manuscript:

LED	Light Emitting Diode
LoRaWAN	Long-Range Wide-Area Network
P&Q	Perturbation and Observation
MQTT	Message Queuing Telemetry Transport
IoT	Internet of Things
HPS	High-Pressure Sodium
LDR	Light-dependent resistors
IR	Infrared Sensors
WiMAX	Worldwide Interoperability for Microwave Access
GSM	Global System for Mobile Communications
LoRa	Long Range
NB-IoT	Narrowband-Internet of Things
WiFi	Wireless Fidelity
BLE	Bluetooth
LTE	Long-Term Evolution
GPRS	General Packet Radio Service
ARM	Advanced RISC Machine
DC	Direct Current
PWM	Pulse Width Modulation
MPPT	Maximum Power Point Tracking
MPP	Maximum Power Point
PI Control	Proportional Integral Control
